# High-intensity exercise in the evening does not disrupt sleep in endurance runners

**DOI:** 10.1007/s00421-019-04280-w

**Published:** 2019-12-07

**Authors:** Craig Thomas, Helen Jones, Craig Whitworth-Turner, Julien Louis

**Affiliations:** 1 Research Institute for Sport and Exercise Sciences (RISES), Liverpool John Moores University, Tom Reilly Building, Byrom St. Campus, Liverpool, L3 3AF UK; 2 Philadelphia 76ers training complex, 1-99 S Front St, Camden, NJ 08103 USA

**Keywords:** Running, Polysomnography, Actigraphy, Heart rate, Heart rate variability

## Abstract

**Purpose:**

To investigate the effect of early evening exercise training at different intensities on nocturnal sleep and cardiac autonomic activity in endurance-trained runners.

**Methods:**

Eight runners completed three experimental trials in a randomised, counterbalanced order. In the early evening (end of exercise 3.5 h before bedtime), participants performed either: (i) a 1 h high-intensity interval running session (HIGH, 6 × 5 min at 90% VO_2peak_ interspersed with 5 min recovery); (ii) a 1 h low-intensity running session (LOW, 60 min at 45% VO_2peak_) or (iii) no exercise (CON). Subsequent nocturnal sleep was assessed using polysomnography, wristwatch actigraphy, and subjective sleep quality. A two-lead electrocardiogram recorded nocturnal cardiac autonomic activity.

**Results:**

Total sleep time increased after HIGH (477.4 ± 17.7 min, *p* = 0.022) and LOW (479.6 ± 15.6 min, *p* = 0.006) compared with CON (462.9 ± 19.0 min). Time awake was lower after HIGH (31.8 ± 18.5 min, *p* = 0.047) and LOW (30.4 ± 15.7 min, *p* = 0.008) compared with CON (46.6 ± 20.0 min). There were no differences between conditions for actigraphy and subjective sleep quality (*p* > 0.05). Nocturnal heart rate variability was not different between conditions, but average nocturnal heart rate increased after HIGH (50 ± 5 beats min^−1^) compared with LOW (47 ± 5 beats min^−1^, *p* = 0.02) and CON (47 ± 5 beats min^−1^, *p* = 0.028).

**Conclusion:**

When performed in the early evening, high-intensity exercise does not disrupt and may even improve subsequent nocturnal sleep in endurance-trained runners, despite increased cardiac autonomic activity. Additionally, low-intensity exercise induced positive changes in sleep behaviour that are comparable to those obtained following high-intensity exercise.

## Introduction

Elite athletes identify sleep as essential for recovery (Fallon 2007; Venter 2014). Indeed, during sleep many bodily processes occur, which are thought to be important for both physical and psychological restoration (Venter 2012). As such, elite athletes are encouraged to maintain good sleep hygiene especially during periods of high training load or competition (Davenne 2009). A minimum of 7 h of sleep per night with at least 85% of sleep efficiency (i.e., time asleep as a percentage of time in bed) is classically recommended (Ohayon et al. 2017; Hirshkowitz et al. 2015). However, many studies have reported an alteration of sleep duration and quality in athletes (e.g., Sargent et al. 2016; Shearer et al. 2015). In a recent meta-analysis, Roberts et al. (2019) reported that elite athletes often did not achieve total sleep recommendations during both training (in 56% and 43% of studies for total sleep time and sleep efficiency, respectively) and competition (in 78% and 62% of studies for total sleep time and sleep efficiency, respectively). It was suggested that the main factors contributing to the worsening of sleep patterns in athletes, albeit evidence was mainly derived from team sports, are training and competition schedules (i.e., early and/or late exercise), travel departure times, jet lag, altitude, and training load. In endurance sports, similar sleep alterations were reported in response to increased training demands (Jurimae et al. 2004; Teng et al. 2011). Teng et al. (2011) measured sleep of elite road cyclists via wristwatch actigraphy during a “baseline phase”, “high-intensity phase”, and a “taper phase”. They showed that sleep duration (7.0 vs. 7.3 h) and sleep efficiency (84.27 vs. 86.25%) were lower during the high-intensity phase compared to baseline. Furthermore, sleep may be impaired to a greater extent when endurance athletes do not adapt well to the training load (Hausswirth et al. 2014; Schaal et al. 2015). In overreached triathletes, Hausswirth et al. (2014) reported a reduction in sleep duration (6:36 vs. 7:09 h), sleep efficiency (88.4 vs. 90.0%), and immobile time (387 vs. 417 min) compared to baseline, before returning to baseline during the taper phase. These findings may suggest high-intensity training or a high volume of training leads to sub-optimal sleep and recovery in endurance athletes.

The time of day that exercise is performed may also influence the sleep response to exercise training. Current sleep hygiene guidelines advise against high-intensity exercise in the evening as opposed to earlier in the day; for the reason, it may increase arousal upon bedtime and impair subsequent night’s sleep (American Sleep Association 2019). Although experimental evidence in non-athletic populations indicates this may be dependent upon the time elapsed between performing high-intensity exercise and bedtime (Stutz et al. 2019). Studies conducted within non-athletic individuals have shown that high-intensity exercise does not disrupt nocturnal sleep (Myllymaki et al. 2011, 2012) and may improve variables such as sleep efficiency, wake after sleep onset, slow-wave sleep (SWS), and non-rapid eye movement (NREM) sleep when performed 2–4 h before bedtime (Dworak et al. 2008; Flausino et al. 2012; Larsen et al. 2019). Whereas disturbed nocturnal sleep (i.e., longer sleep onset latency) has only been observed after high-intensity exercise performed 1 h before bedtime (Oda and Shirakawa 2014). In contrast to this norm, Ramos-Campo et al. (2019) recently compared sleep of amateur ultra-endurance runners using wristwatch actigraphy, after a 60 min run at 60% VO_2max_ and after vigorous exercise consisting of 7 × 3 min runs at 100% VO_2max_, in the early evening. Both sleep efficiency (88.2 vs. 84.3%) and the average time of awakening (2.3 vs. 3.0 min) were improved following the moderate-intensity run compared with the vigorous run, without a concomitant change in heart rate (HR) or heart rate variability (HRV). This study seemed to indicate that vigorous exercise performed in the early evening should be avoided to prevent affecting sleep in endurance athletes. Nonetheless, these researchers did not report bedtime or standardise time in bed, nor was there a control included or a measure of polysomnography, which is the gold standard measurement for sleep. Additionally, most of the studies investigating the effects of evening exercise in trained individuals have been conducted in team sports following an evening match. In these observational studies, bedtime and get-up time was also not standardised, thus very likely influencing sleep variables. For example, in the study by Nédélec et al. (2019), mean bedtime (02:19 am) was delayed by 107 min after evening matches compared to day training in professional soccer players. However, get-up time the next morning did not change due to travel constraints, which could largely explain the reduction in total sleep time (− 92 min) and sleep efficiency (− 4%), rather than exercise intensity alone. Hence, the influence of exercise intensity when training is performed in the evening is still unclear. Besides, the paucity of data on sleep of endurance athletes warrants more research within this population.

Therefore, the aim of the current study was to investigate whether exercise of differing intensities in the early evening would elicit changes in subsequent nocturnal sleep and cardiac autonomic activity within a group of endurance-trained runners. Such information could assist with optimising training prescription to improve the recovery and exercise performance of endurance athletes. Based on previous findings in endurance athletes, albeit limited, it was hypothesised that high-running, would disturb nocturnal sleep and cardiac autonomic activity in comparison to low-intensity running and a no-exercise control.

## Materials and methods

### Participants

Eight endurance-trained male runners (age = 27.8 ± 6.9 years; height = 1.8 ± 0.1 m; body weight = 73.5 ± 5.3 kg; peak oxygen uptake = 57 ± 4 ml·kg^−1^ min^−1^ and 10 k personal best = 38:06 ± 02:00 mm:ss) were recruited from amateur running clubs. The inclusion criteria consisted of 18–40 years of age, a 10-km race time ≤ 41 min, running experience of at least 3 years, training frequency of ≥ 4 days, and a weekly-accumulated running distance ≥ 15 miles. Athletes were classed in performance level 3 according to guidelines for participant’s classification in sports science research (De Pauw et al. 2013). None of the participants used sleep medication, were working night shifts, or had travelled across different time zones in the month prior to selection, but were familiar with training in the early evening. Before commencing the study, a full explanation of the protocol and associated risks was provided, and written informed consent was obtained. In addition, participants completed the Pittsburgh Sleep Quality Index (PSQI) (Buysse et al. 1989) and the composite morningness, sleep flexibility/rigidity (*F*/*R*) and languid/vigour (*L*/*V*) (Smith et al. 1989) questionnaires for the assessment of sleep quality, chronotype, and other personal attributes. Mean PSQI score was 3 ± 1 and mean chronotype score was 37.3 ± 4.1, all intermediate types; *F*/*R* score, 49.8 ± 0.5; and *V*/*L* score, 36.0 ± 5.5. This study was ethically approved from the Local University research ethics committee.

### Experimental design

Participants made two preliminary visits consisting of a cardio-respiratory fitness test, followed by a familiarisation night with polysomnography in the sleep laboratory, in an attempt to prevent any first night effects (Agnew et al. 1966). High-intensity interval running (HIGH, 6 × 5 min at 90% VO_2peak_ interspersed with 6 × 5 min at 60% VO_2peak_), low-intensity running (LOW, 60 min at 45% VO_2peak_), and no exercise (CON) were then performed in a randomised, counterbalanced order, separated by a minimum of 4 days. Subsequent overnight sleep was measured via polysomnography, wristwatch actigraphy, and subjective sleep quality scale. Nocturnal cardiac autonomic activity was recorded using a two-lead electrocardiogram as part of the polysomnography setup. In the 48 h prior to the experimental trials, participants abstained from strenuous exercise and maintained their normal sleeping patterns. In the 24 h before, participants self-recorded their habitual diet and repeated it prior to subsequent trials. On the day of each experimental trial, no caffeinated products were permitted 12 h before sleep and a standardised meal (2 g CHO kg^−1^ body mass (BM), 0.08 g FAT kg^−1^BM and 0.12 g PRO kg^−1^BM) was consumed at 4 pm.

### Assessment of cardio-respiratory fitness

For the assessment of peak oxygen uptake (VO_2peak_), an incremental exercise test was performed on a motorised treadmill (HP Cosmos, Germany). A gas analysis system (Oxycon Pro, Carefusion, Germany) was calibrated beforehand and used to continuously measure oxygen uptake (VO_2_) during the exercise protocol. The test was completed in 2 min stages, beginning at 8 km h^−1^, which increased to 10 km h^−1^, 12 km h^−1^, 14 km h^−1^, and 16 km h^−1^. On completion of the 16 km h^−1^ stage, the treadmill gradient was increased by 1% every 2 min until volitional exhaustion. VO_2peak_ was considered to be achieved by the following endpoint criteria: (i) HR within 10 beats min^−1^ of age predicted maximum (ii) a maximum rating of perceived exertion (RPE) (Borg 1970), (iii) a respiratory exchange ratio > 1.1, and (iv) a plateau in VO_2_ despite an increase in workload (American College of Sports Medicine 2014). To determine the peak VO_2_ of each stage, a mean from the last 30 s of each 2 min was recorded. Using the regression of VO_2_ and velocity, running speeds corresponding to 45%, 60%, and 90% of VO_2peak_ were then calculated.

### Experimental protocol

Exercise sessions commenced at 5:50 pm with a 10 min self-selected warm up followed by 1 h of running on a motorised treadmill in a temperature-controlled laboratory (20–22 °C). To monitor the physiological response to exercise, RPE was indicated on the Borg scale (6–20) every 15 min and HR was recorded via telemetry (FT1, Polar, Kempele, Finland) every 5 min. On the evening of the CON, participants completed no exercise and remained seated for 1 h in a rested state. After, participants were allowed to shower with a standardised duration (HIGH = 04:25 ± 01:52 mm:ss; LOW = 04:28 ± 01:51 mm:ss; CON = 04:23 ± 01:52 mm:ss) and water temperature (HIGH = 35.3 ± 3.3 °C; LOW = 35.4 ± 3.4 °C; CON = 35.3 ± 3.3 °C) for each overnight stay. To retrieve this information, participants documented the duration from a stopwatch on their mobile phone and recorded the water temperature using a digital temperature probe (Electronic Temperature Instruments, Thermamite, UK) placed underneath the showerhead. Once showered, participants ate the same evening snack (2 × pieces of toasted white bread with butter and strawberry jam) and were allowed to drink water ad libitum (HIGH = 1368 ± 325 ml; LOW = 994 ± 241 ml; CON = 667 ± 366 ml).

At approximately 7.45 pm, participants were shown to the sleep laboratory where they were prepped for overnight polysomnography. The room used was dark, quiet and maintained between the ambient temperatures of 20.5 °C and 21.5 °C. During the preparation period, participants sat in a chair and only got up if they needed a toilet break. The use of electronic devices, i.e., mobiles and personal computers, were also not permitted to avoid blue light exposure. After polysomnography preparation was completed, participants were instructed to wear an actiwatch (Actiwatch 4, Cambridge Neurotechnology Ltd, UK) on their non-dominant wrist and check that their mobile phone was turned off. They then laid awake in a single bed (Contoura 480, Huntleigh Technology, Hong Kong) underneath a medium tog duvet, in which their nightwear and pillow setup was the same as they had used during the familiarisation night. An impedance check of the polysomnography channels was performed before lights out to ensure levels were between 0 and 4 kOhm, a range considered low by the manufacturing company. Bedtime for participants was imposed at 10:30 pm and get-up time was at 7 a.m. the next morning. Following the overnight sleep, the electrodes and actiwatch were removed, and participants were asked to provide their subjective sleep quality before they were free to start their day.

## Measurements

### Polysomnography

A SOMNOscreen plus (SOMNOmedics, Germany) was used for overnight polysomnography. The set up included a three-channel electroencephalogram (EEG) (F3-M2, C3-M2 and O1-M2), two-channel electrooculogram (EOG) (E1-M2 and E2-M1), submental chin electromyogram (EMG)—for the assessment of sleep architecture; and a two-lead electrocardiogram (ECG)—for the assessment of cardiac autonomic activity. EEG, EOG and EMG electrodes were attached with electrode cream (Grass, EC2 cream) and electrode glue (Mavidon, Collodion glue). EEG sites were located using the international 10–20 system. The signals were recorded at a sampling rate of 256 fps within a frequency range of 0.2–128 Hz. To conduct the ECG two monitoring electrodes were attached to the torso in parallel to the right shoulder and left hip. All raw physiological data were transmitted from the polysomnography head box to a personal computer via a Bluetooth data receiver.

Scoring of sleep stages was automatically processed by a sleep software package (DOMINO version 2.8.0, SOMNOmedics, Germany) and subsequently visually inspected in 30 s epochs using the standard criteria from the American Academy of Sleep Medicine (Iber et al. 2007). Visual inspection was carried out by the lead experimenter who was trained in scoring sleep by a respiratory and sleep physiologist. The stages scored included wakefulness, NREM sleep stage 1, NREM sleep stage 2, SWS, and rapid eye movement (REM) sleep. From this analysis, the sleep variables used were: time in bed (min), total sleep time (min), sleep onset latency—stage 1 (min), sleep onset latency—stage 2 (min), deep sleep latency (min), REM latency (min), time awake (min), NREM stage 1 (min), NREM stage 2 (min), SWS (min), REM (min), NREM stage 1 (%), NREM stage 2 (%), SWS (%), and REM (%). The electrocardiogram provided two indices of cardiac autonomic activity: average nocturnal HR and average nocturnal standard deviation of the *R*-to-*R* interval (SDRR), which is a time-based measure of HRV (Shaffer and Ginsberg 2017).

### Wristwatch actigraphy

Actigraphy was used as it provides movement-related information of sleep behaviour (Ancoli-Israel et al. 2003). The actiwatch was set to an epoch length of 1 min and at a medium sensitivity. As bedtime was known, participants wore the actiwatch through the night and pressed the marker button for 2–3 s upon their final awakening the next morning. The Consensus Sleep Diary (Carney et al. 2012) was then filled in, which asks questions relating to sleep latency, wake-up time, and the number of awakenings. Both the actiwatch and the Consensus Sleep Diary were used to determine sleep onset and wake-up time, so that sleep behaviour could be automatically calculated using the appropriate actiwatch software (Actiwatch activity and sleep analysis version 5.24, Cambridge Neurotechnology Ltd, UK). From the actiwatch analysis, the following four sleep variables were used: total sleep time (min), sleep onset latency (min), fragmentation index (restlessness), and total activity score (number of activity counts).

### Subjective sleep quality

The Likert scale from the Consensus sleep diary was used to obtain a rating of subjective sleep quality the morning after the overnight sleep. Within an hour of being awake, participants answered the question “How would you rate your overall sleep quality?” with a scale ranging from 1–5 (1 = very poor; 2 = poor; 3 = fair; 4 = good; 5 = very good). No discussion was made with the participant and they were allowed as much time as they wanted to respond.

### Statistical analysis

Statistical Package for the Social Sciences (SPSS v25) was used for data analysis. Data were checked for normality and are presented as mean ± standard deviation. A one-way repeated-measures ANOVA was performed to compare parameters from polysomnography, actigraphy, and the electrocardiogram between conditions. The non-parametric Friedman test was used to assess subjective sleep quality from the Likert scale. Where a significant ANOVA main effect was identified, post hoc analyses were conducted using the least significant difference (LSD) method. Greenhouse–Geisser values were used if the assumption of Sphericity was not met. Effect size (Cohen’s *d*) of all significant differences was calculated by dividing the difference in group means by the pooled standard deviation and was assessed using the following thresholds: < 0.20 = trivial effect; 0.20–0.60 = small effect; > 0.60–1.20 = moderate effect; > 1.20–2.00 = large effect; > 2.00–4.00 = very large; > 4.00 = extremely large (Hopkins et al. 2009). Statistical significance was set at level *P* < 0.05.

## Results

### Physiological and perceptual responses to exercise

In the HIGH condition, running speed was 15.6 ± 0.3 km h^−1^ at 90% VO_2peak_ and 10.4 ± 0.7 km h^−1^ at 60% VO_2peak_. HR incrementally increased with moderate- and high-intensity running. Maximum HR was 149 ± 14 beats min^−1^ during moderate-intensity stages and 176 ± 12 beats min^−1^ during high-intensity stages. RPE at the end of the run was 18 ± 1 AU (very hard). In the LOW condition, running speed was 7.9 ± 0.9 km h^−1^ at 45% VO_2peak_. HR reached a maximum value of 114 ± 6  beats min^−1^ and RPE at the end of the run was 11 ± 1 AU (fairly light).

### Polysomnography, actigraphy and subjective sleep quality

**Fig. 1:**
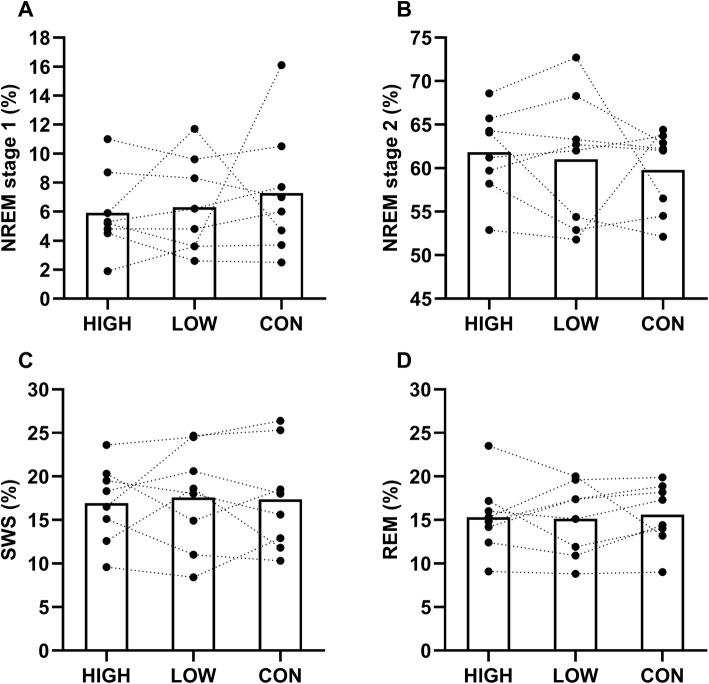
Individual responses for NREM stage 1 (**a**), NREM stage 2 (**b**) SWS (**c**), and REM (**d**) after different running intensities and CON. White bars are the mean values of each sleep parameter and the black markers and dashed lines represent the individual values

Sleep variables from overnight polysomnography are shown in Table 1. Time in bed was not different between conditions. Exercise had a significant main effect on total sleep time (*p* = 0.005) and time awake (*p* = 0.016). Total sleep time was longer after HIGH [477.4 ± 17.7 min, *p* = 0.022, ES 0.79 (moderate effect)] and LOW [479.6 ± 15.6 min, *p* = 0.006, ES 0.96 (moderate effect)] compared with CON (462.9 ± 19.0 min). Time awake was lower after HIGH [31.8 ± 18.5 min, *p* = 0.047, ES 0.77 (moderate effect)] and LOW [30.4 ± 15.7 min, *p* = 0.008, ES 0.90 (moderate effect)] compared with CON (46.6 ± 20.0 min). There was no difference between HIGH and LOW for total sleep time (477.4 ± 17.7 vs. 479.6 ± 15.6 min, *p* = 0.641) and time awake (31.8 ± 18.5 vs. 30.4 ± 15.7 min, *p* = 0.801). No exercise effect was found for sleep onset latency stage 1, sleep onset latency stage 2, deep sleep latency, REM latency, NREM stage 1, NREM stage 2, SWS, and REM (*p* > 0.05). Individual responses after exercise and no exercise for each sleep stage are shown in Fig. 1. There were also no differences between conditions for actigraphy variables and subjective sleep quality (*p* > 0.05) (Table 2).

**Table 1:** Sleep variables from polysomnography after different running intensities and CON

	HIGH	LOW	CON
Time in bed (min)	510 ± 0	510 ± 0	510 ± 0
Total sleep time (min)	477.4 ± 17.7*	479.6 ± 15.6*	462.9 ± 19.0
Sleep onset latency S1 (min)	11.8 ± 8.8	7.8 ± 4.4	12.1 ± 7.7
Sleep onset latency S2 (min)	14.4 ± 10.4	9.5 ± 4.5	14.9 ± 7.0
Deep sleep latency (min)	33.7 ± 12.9	24.3 ± 9.2	28.2 ± 6.4
REM latency (min)	101.6 ± 43.5	99.8 ± 40.5	102.0 ± 33.0
Time awake (min)	31.8 ± 18.5*	30.4 ± 15.7*	46.6 ± 20.0
NREM stage 1 (min)	28.0 ± 12.5	30.0 ± 15.1	33.3 ± 19.2
NREM stage 2 (min)	295.8 ± 31.4	293.2 ± 42.1	276.7 ± 24.8
SWS (min)	81.0 ± 22.2	84.0 ± 27.9	80.3 ± 27.9
REM (min)	72.7 ± 17.3	72.5 ± 19.7	72.7 ± 18.7
NREM stage 1 (%)	5.9 ± 2.8	6.3 ± 3.3	7.3 ± 4.3
NREM stage 2 (%)	61.8 ± 4.9	61.0 ± 7.5	59.8 ± 4.7
SWS (%)	16.9 ± 4.5	17.6 ± 5.9	17.4 ± 6.0
REM (%)	15.3 ± 4.1	15.1 ± 4.2	15.6 ± 3.6

^*^Indicates *p* < 0.05 compared to CON (*n* = 8)

**Table 2:** Sleep variables from wristwatch actigraphy and subjective sleep quality after different running intensities and CON (*n* = 8)

	HIGH	LOW	CON
Total sleep time (min)	457 ± 18	453 ± 17	442 ± 17
Sleep onset latency (min)	9 ± 3	7 ± 4	8 ± 3
Fragmentation index (%)	26.8 ± 9.2	24.4 ± 9.2	30.7 ± 13.7
Total activity score (count)	4734 ± 2531	4653 ± 2220	5950 ± 2872
Subjective sleep quality (1–5)	2.88 ± 1.13	3.25 ± 0.89	3.38 ± 1.06

### Electrocardiogram

**Fig. 2:**
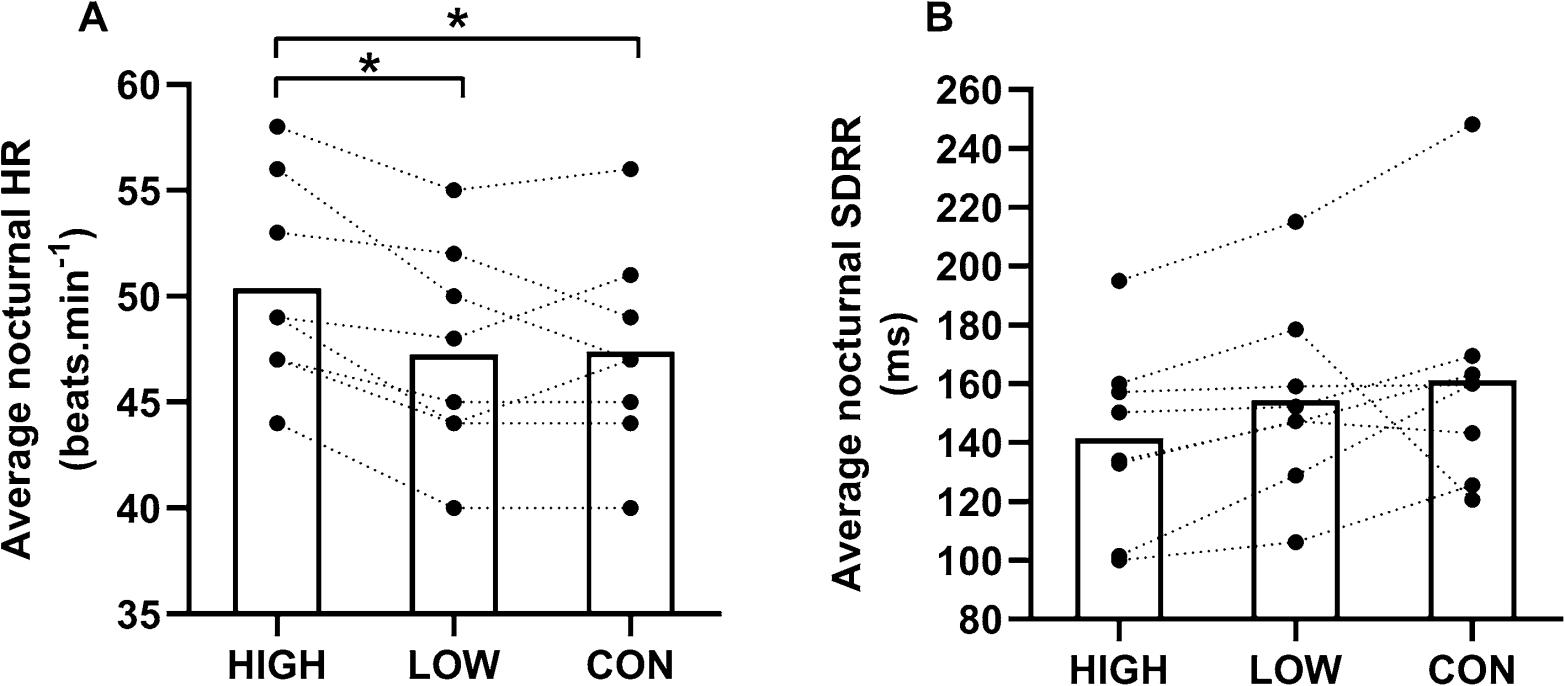
Average nocturnal HR (**a**) and average nocturnal SDRR (**b**) after different running intensities and CON. *Indicates *p* < 0.05. White bars are the mean values and the black markers and dashed lines represent the individual values

A graphical representation of average nocturnal HR and average nocturnal SDRR is presented in Fig. 2. There was a significant main effect of exercise on average nocturnal HR (*p* = 0.004). HIGH increased average nocturnal HR (50 ± 5 beats min^−1^) compared to LOW [47 ± 5  beats min^−1^, *p* = 0.002, ES 1.73 (large effect)] and CON (47 ± 5 beats min^−1^, *p* = 0.028, ES 0.98 (moderate effect)]. No difference in average nocturnal HR was found between LOW and CON (47 ± 5 beats min^−1^ vs. 47 ± 5 beats min^−1^, *p* = 0.882). There was no difference observed between conditions for average nocturnal SDRR (*p* = 0.146).

## Discussion

Using a randomised, counterbalanced design, the current study investigated whether exercise of differing intensities (HIGH vs. LOW vs. CON) in the early evening would elicit changes in nocturnal sleep and cardiac autonomic activity within a group of endurance-trained runners. Contrary to our initial hypothesis, total sleep time increased, whilst time awake decreased in a similar proportion after both HIGH and LOW compared to CON. HIGH also increased average nocturnal HR, but did not alter nocturnal HRV compared to LOW and CON. These results contrast current sleep hygiene guidelines and suggest that endurance athletes may perform high-intensity training in the early evening with no disruption to nocturnal sleep.

### Effect of exercise intensity on subsequent night’s sleep

One novel aspect of the current study is the measurement of sleep in endurance-trained individuals using polysomnography, following different exercise intensity protocols. Our data suggest that total sleep time increased after HIGH compared to CON, indicating high-intensity exercise may acutely increase sleep duration in endurance athletes. Current sleep hygiene guidelines advise against high-intensity exercise in the evening due to concerns it may impair sleep (American Sleep Association 2019), our data do not support these guidelines. Since time in bed was standardised in all experimental and control conditions, we are confident that the increased time asleep was due to the reduction in time awake and not factors surrounding sleep scheduling. Using wristwatch actigraphy, Ramos-Campo et al. (2019) reported that moderate-intensity exercise improved sleep efficiency and average time of awakening compared to vigorous intensity exercise in amateur ultra-endurance runners. However, these researchers did not report bedtime or standardise time in bed and they did not include a control condition or use polysomnography. In this study, the sleep changes observed after high-intensity exercise are comparable to other studies using polysomnography in non-athletic populations. For example, both sleep efficiency and wake after sleep onset were improved after moderate- and high-intensity running, 2 h before bedtime, in healthy men (Flausino et al. 2012). Another study in children demonstrated sleep efficiency and SWS percentage were increased after high but not moderate-intensity cycling, 3–4 h before bedtime (Dworak et al. 2008). In contrast, Oda and Shirakawa (2014) found a reduction in total sleep time and sleep efficiency in physically active men, after high-intensity running, although exercise was performed 1 h before bedtime in this study rather than in the early evening in the current and aforementioned studies. Previously, observational studies have indicated that either the volume or intensity of training may lead to sub-optimal sleeping patterns and recovery in endurance athletes, though these studies did not control training session times nor bedtime (Hausswirth et al. 2014; Jurimae et al. 2004; Schaal et al. 2015; Teng et al. 2011). This was recently confirmed by Roberts et al. (2019), reporting that large increases in training load (> 25%) impaired sleep in rugby players, synchronised swimmers, and cyclists. Lastella et al. (2018) even suggested that disturbances in sleep might represent an indicator of overreaching/overtraining in athletes. Conversely, our findings suggest that high-intensity exercise performed acutely in the early evening (ending 3.5 h before bedtime) does not impair and may even improve sleep in endurance athletes. However, the effects of chronic endurance training in the early evening on sleep remain to be investigated.

Another interesting finding was that total sleep time increased, whilst time awake decreased in a similar proportion after performing both HIGH and LOW compared with CON. This suggests endurance athletes could complete either a high-intensity session or a low-intensity session interchangeably without risking disturbance to nocturnal sleep. Such data are similar to that of Horne and Staff (1983) who, in physically trained runners, found low- and high-intensity exercise in the afternoon increased total sleep time and SWS percentage, respectively. More recently, studies in sedentary individuals observed that high-intensity exercise in the evening had a beneficial effect on nocturnal sleep compared with low-intensity exercise (Hayashi et al. 2014; Wong et al. 2013). The exact mechanism by which exercise training improves sleep is unclear (Driver and Taylor 2000), though increased energy expenditure or the decline in body temperature from increased peripheral skin blood flow are suggested to be involved (Driver and Taylor 2000). Even though it was not directly measured in our protocol, energy expenditure in HIGH was likely higher than LOW and did not have an additional benefit on total sleep time in the subsequent night. It is also unlikely that body temperature fluctuations following exercise would have influenced the change in nocturnal sleep. The increase in body temperature with exercise has been shown to return to baseline levels within 45–90 min after exercise is terminated, depending on the intensity that is performed (Horne and Staff 1983; Miller et al. 2019). In the current study, it is assumed that body temperature would have been similar upon bedtime after both exercise sessions and the control condition due to the 3.5 h recovery period that followed. One possible explanation for our results might be that exercise caused a phase advance of the human circadian pacemaker, independent of intensity. Both the onset and peak phase of plasma melatonin have been shown to occur earlier after a single bout of exercise, whereas this did not change with no exercise (Buxton et al. 2003; Miyazaki et al. 2001). Future investigations should extend the analysis to more variables (i.e., energy expenditure, body temperature, melatonin, and adenosine) that could help to understand the underlying mechanism(s) of increased sleep duration after early evening exercise.

### Effect of exercise intensity on nocturnal cardiac autonomic activity

The measurement of cardiac autonomic activity during the night was another novel aspect. Usually, in the transition from wake to sleep, parasympathetic activity is high, as shown by a reduction in HR and an increase in HRV (Trinder et al. 2001). Nevertheless, in the current study, following HIGH, though there was no alteration in HRV, nocturnal HR was increased compared to LOW and CON, suggesting there was a greater contribution from the sympathetic nervous system. This is in contrast to Ramos-Campo et al. (2019) who concluded that there was no change in nocturnal HR or HRV in amateur ultra-endurance runners after moderate and vigorous intensity exercise. Despite the increase in nocturnal HR, there was no disruption to sleep behaviour in the current study. This opposes the idea that increased arousal upon bedtime disturbs nocturnal sleep (American Sleep Association 2019). Various physiological changes depend upon exercise intensity may explain the delayed recovery of sympathetic activity, including the restoration of energy reserves, increased catecholamine release, and the clearance of lactate, as they all increase post-exercise oxygen consumption (Frey et al. 1993; Gaesser and Brooks 1984). The changes in cardiac autonomic activity recorded after HIGH are in agreement with the previous studies in physically active individuals showing an increase in nocturnal HR but not HRV after moderate- and high-intensity exercise in the evening (Myllymaki et al. 2011, 2012). There was also no corresponding change in sleep patterns measured via actigraphy in these studies (Myllymaki et al. 2011, 2012). Thus, an increase in nocturnal HR following evening exercise would not necessarily disrupt sleep duration and quality. Moreover, the monitoring of nocturnal HRV may not be sensitive to detecting changes in cardiac autonomic homeostasis after exercise training.

### Practical implications and limitations

The current study observed that total sleep time within endurance-trained runners was increased after an acute bout of high- and low-intensity exercise in the early evening compared with no exercise. In contrast with current sleep hygiene guidelines (American Sleep Association 2019), our data suggest that endurance athletes may perform high-intensity training in the early evening with no deleterious consequences on nocturnal sleep. Interestingly, this study also demonstrated that low-intensity training in the early evening had a similar effect on nocturnal sleep but without disturbing cardiac autonomic homeostasis. Scheduling low-intensity training in the early evening could be advantageous if there is another training session planned the next morning due to the increase in total sleep time. Coaches and athletes could use these findings to help facilitate the balance between training stress and recovery to enhance the adaptation process. Although we used the gold standard polysomnography to measure nocturnal sleep, it is important to consider the limitations of the study. The participants were asked to sleep in an unfamiliar environment for several nights (including a familiarisation night) in the sleep laboratory, which could alter sleep patterns compared to sleeping at home. Body temperature, energy expenditure, melatonin, and humoral substances (i.e., adenosine) related to sleep pressure were not measured, so the mechanism(s) explaining our results are unclear. It is also unknown from this study whether chronic exercise training in the evening may affect nocturnal sleep as well as the effects of training at other times of the day. Future research should examine if exercise training closer to bedtime may disrupt subsequent nocturnal sleep and the potential influence of substrate availability and muscle damage.

## Conclusions

In conclusion, the current study demonstrates that high-intensity exercise when performed in the early evening (ending 3.5 h before bedtime) does not disturb and may even improve subsequent night’s sleep in endurance runners despite increased cardiac autonomic activity. These data contrast with current sleep hygiene guidelines for performing exercise in the evening. Furthermore, low-intensity exercise performed at the same time of day induces positive changes in sleep behaviour that are comparable to high-intensity exercise.

## Abbreviations

ANOVA: Analysis of variance
BM: Body mass
CHO: Carbohydrate
CON: No exercise
ECG: Electrocardiogram
EEG: Electroencephalogram
EMG: Electromyogram
EOG: Electrooculogram
HIGH: High-intensity interval running
HR: Heart rate
HRV: Heart rate variability
LOW: Low-intensity running
LSD: Least significant difference
NREM: Non-rapid eye movement sleep
PRO: Protein
PSQI: Pittsburgh sleep quality index
REM: Rapid eye movement sleep
RPE: Rating of perceived exertion
SDRR: Standard deviation of the *R*-to-*R* interval
SWS: Slow-wave sleep
VO_2_: Oxygen uptake
VO_2peak_: Peak oxygen uptake

## References

[CR1] Agnew HW, Webb WB, Williams RL (1966). The first night effect: an EEG study of sleep. Psychophysiology.

[CR2] American College of Sports Medicine (2014) Health related physical fitness testing and interpretation. In: ACSM’s guidelines for exercise testing and prescription (9th ed). Lippincott, Williams and Wilkins, Baltimore (MD), pp 72-93

[CR3] American Sleep Association (2019) Sleep hygiene tips. https://www.sleepassociation.org/about-sleep/sleep-hygiene-tips/. Accessed 2nd May 2019.

[CR4] Ancoli-Israel S, Cole R, Alessi C, Chambers M, Moorcroft W, Pollak CP (2003). The role of actigraphy in the study of sleep and circadian rhythms. Sleep.

[CR5] Borg G (1970). Perceived exertion as an indicator of somatic stress. Scand J Rehabil Med.

[CR6] Buxton Orfeu M., Lee Calvin W., L'Hermite-Balériaux Mireille, Turek Fred W., Van Cauter Eve (2003). Exercise elicits phase shifts and acute alterations of melatonin that vary with circadian phase. American Journal of Physiology-Regulatory, Integrative and Comparative Physiology.

[CR7] Buysse DJ, Reynolds CF, Monk TH, Berman SR, Kupfer DJ (1989). The Pittsburgh sleep quality index: a new instrument for psychiatric practice and research. Psychiatry Res.

[CR8] Carney CE, Buysse DJ, Ancoli-Israel S (2012). The consensus sleep diary: standardizing prospective sleep self-monitoring. Sleep.

[CR9] Davenne D (2009). Sleep of athletes: problems and possible solutions. Biol Rhythm Res.

[CR10] De Pauw K, Roelands B, Cheung SS, De Geus B, Rietjens G, Meeusen R (2013). Guidelines to classify subject groups in sport-science research. Int J Sports Physiol Perform.

[CR11] Driver HS, Taylor SR (2000). Exercise and sleep. Sleep Med Rev.

[CR12] Dworak M, Wiater A, Alfer D, Stephan E, Hollmann W, Struder HK (2008). Increased slow wave sleep and reduced stage 2 sleep in children depending on exercise intensity. Sleep Med.

[CR13] Fallon KE (2007). Blood tests in tired elite athletes: expectations of athletes, coaches and sports science/sports medicine staff. Br J Sports Med.

[CR14] Flausino NH, Da Silva Prado JM, De Queiroz SS, Tufik S, De Mello MT (2012). Physical exercise performed before bedtime improves the sleep pattern of healthy young good sleepers. Psychophysiology.

[CR15] Frey GC, Byrnes WC, Mazzeo RS (1993). Factors influencing excess post-exercise oxygen consumption in trained and untrained women. Metabolism.

[CR16] Gaesser GA, Brooks GA (1984). Metabolic bases of excess post-exercise oxygen consumption: a review. Med Sci Sports Exerc.

[CR17] Hausswirth C, Louis J, Aubry A, Bonnet G, Duffield R, Le Meur Y (2014). Evidence of disturbed sleep and increased illness in overreached endurance athletes. Med Sci Sports Exerc.

[CR18] Hayashi Y, Nishihira Y, Higashiura T, Usui S (2014). The effects of different intensities of exercise on night sleep. Adv Exerc Sports Physiol.

[CR19] Hirshkowitz M, Whiton K, Albert SM (2015). National sleep foundation’s sleep time duration recommendations: methodology and results summary. Sleep Health.

[CR20] Hopkins W, Marshall SW, Batterham AM, Hanin J (2009). Progressive statistics for studies in sports medicine and exercise science. Med Sci Sports Exerc.

[CR21] Horne JA, Staff LHE (1983). Exercise and sleep: body-heating effects. Sleep.

[CR22] Iber C, Ancoli-Israel S, Chesson A, Quan S (2007). The AASM manual for the scoring of sleep and associated events: rules, terminology and technical specifications.

[CR23] Jurimae J, Maestu J, Purge P, Jurimae T (2004). Changes in stress and recovery after heavy training in rowers. J Sci Med Sport.

[CR24] Larsen P, Marino F, Melehan K, Guelfi KJ, Duffield R, Skein M (2019). Evening high intensity interval exercise does not disrupt sleep or alter energy intake despite changes in acylated ghrelin in middle-aged men. Exp Physiol.

[CR25] Lastella M, Vincent GE, Duffield R, Roach GD, Halson SL, Heales LJ, Sargent C (2018). Can sleep be used as an indicator of overreaching and overtraining in athletes?. Front Physiol.

[CR26] Miller DJ, Sargent C, Roach GD, Scanlan AT, Vincent GE, Lastella M (2019). Moderate-intensity exercise performed in the evening does not impair sleep in healthy males. Eur J Sport Sci.

[CR27] Miyazaki T, Hashimoto S, Masubuchi S, Honma S, Honma KI (2001) Phase-advance shifts of human circadian pacemaker are accelerated by daytime physical exercise. Am J Physiol Regul Integr Comp Physiol 284(3):714–724 10.1152/ajpregu.2001.281.1.R197 11404294

[CR28] Myllymaki T, Kyrolainen H, Savolainen K (2011). Effects of vigorous late-night exercise on sleep quality and cardiac autonomic activity. J Sleep Res.

[CR29] Myllymaki T, Rusko H, Syvaoja H, Juuti T, Kinnunen ML, Kyrolainen H (2012). Effects of exercise intensity and duration on nocturnal heart rate variability and sleep quality. Eur J Appl Physiol.

[CR30] Nédélec M, Dawson B, Dupont G (2019). Influence of night soccer matches on sleep in elite players. J Strength Condit Res.

[CR31] Oda S, Shirakawa K (2014). Sleep onset is disrupted following pre sleep exercise that causes large physiological excitement at bedtime. Eur J Appl Physiol.

[CR32] Ohayon M, Wickwire EM, Hirshkowitz M (2017). National sleep foundation’s sleep quality recommendations: first report. Sleep Health.

[CR33] Ramos-Campo DJ, Avila-Gandia V, Luque AJ, Rubio-Arias JA (2019). Effects of hour training and exercise intensity on nocturnal autonomic modulation and sleep quality of amateur ultra-endurance runners. Physiol Behav.

[CR34] Roberts S, Teo W-P, Warmington SA (2019). Effects of training and competition on the sleep of elite athletes: a systematic review and meta-analysis. Br J Sports Med.

[CR35] Sargent C, Roach GD (2016). Sleep duration is reduced in elite athletes following night-time competition. Chronobiol Int.

[CR36] Schaal K, Le Meur Y, Louis J (2015). Whole-body cryostimulation limits overreaching in elite synchronized swimmers. Med Sci Sports Exerc.

[CR37] Shaffer F, Ginsberg JP (2017). An overview of heart rate variability metrics and norms. Front Public Health.

[CR38] Shearer DA, Jones RM, Kilduff LP, Cook CJ (2015). Effects of competition on the sleep patterns of elite rugby union players. Eur J Sport Sci.

[CR39] Smith CS, Reilly C, Midkiff K (1989). Evaluation of three circadian rhythm questionnaires with suggestions for an improved measure of morningness. J Appl Psychol.

[CR40] Stutz J, Eiholzer R, Spengler CM (2019). Effects of evening exercise on sleep in healthy participants: a systematic review and meta-analysis. Sports Med.

[CR41] Teng E, Lastella M, Roach GD, Sargent C (2011). The effect of training load on sleep quality and sleep perception in elite male cyclists. Kennedy GA, Sargent C Little clock, big clock: molecular to physiological clocks.

[CR42] Trinder J, Kleiman J, Carrington M (2001). Autonomic activity during human sleep as a function of time and sleep stage. J Sleep Res.

[CR43] Venter RE (2012). Role of sleep in performance and recovery of athletes: a review article. South Afr J Res Sport Phys Edu Recreat.

[CR44] Venter RE (2014). Perceptions of team athletes on the importance of recovery modalities. Eur J Sports Sci.

[CR45] Wong SN, Halaki M, Chow CM (2013). The effects of moderate to vigorous aerobic exercise on the sleep need of sedentary young adults. J Sports Sci.

